# Application of metagenomic next-generation sequencing for rapid molecular identification in spinal infection diagnosis

**DOI:** 10.3389/fcimb.2024.1382635

**Published:** 2024-07-01

**Authors:** Hui Lv, Sheng Liao, Zhenzhen Shi, Yuan Guo, JianHong Zhou, Hui Chen, Fei Luo, JianZhong Xu, ZhongRong Zhang, ZeHua Zhang

**Affiliations:** ^1^ Department of Spine Surgery, Jiangbei Branch of Southwest Hospital, 958th Hospital of the PLA Army, Chongqing, China; ^2^ Department of Orthopaedic, Southwest Hospital, The First Affiliated Hospital of Army Medical University, Chongqing, China; ^3^ Department of Medecine, Dinfectome Inc., Nanjing, Jiangsu, China

**Keywords:** spinal infection, metagenomic next-generation sequencing, targeted next-generation sequencing, spinal tuberculosis, sensitivity, diagnosis

## Abstract

**Objective:**

This study aimed to determine the sensitivity and specificity of metagenomic next−generation sequencing (mNGS) for detecting pathogens in spinal infections and to identify the differences in the diagnostic performance between mNGS and targeted next−generation sequencing (tNGS).

**Methods:**

A total of 76 consecutive patients with suspected spinal infections who underwent mNGS, culture, and histopathological examinations were retrospectively studied. The final diagnosis of the patient was determined by combining the clinical treatment results, pathological examinations, imaging changes and laboratory indicators. The sensitivity and specificity of mNGS and culture were determined.

**Results:**

The difference between the two detection rates was statistically significant (*p* < 0.001), with mNGS exhibiting a significantly higher detection rate (77.6% versus 18.4%). The average diagnosis time of mNGS was significantly shorter than that of bacterial culture (*p* < 0.001, 1.65 versus 3.07 days). The sensitivity and accuracy of mNGS were significantly higher than that of the culture group (*p* < 0.001, 82.3% versus 17.5%; 75% versus 27.6%), whereas the specificity of mNGS (42.9%) was lower than that of the culture group (*p* > 0.05, 42.9% versus 76.9%). The sensitivity, specificity, accuracy, and positive predictive value (PPV) of pus were higher than those of tissue samples for mNGS, whereas for culture, the sensitivity, specificity, accuracy, and PPV of tissue samples were higher than those of pus. tNGS demonstrated higher sensitivity and accuracy in diagnosing tuberculosis (TB) than mNGS (80% versus 50%; 87.5% versus 68.8%).

**Conclusion:**

mNGS for spinal infection demonstrated better diagnostic value in developing an antibiotic regimen earlier, and it is recommended to prioritize pus samples for testing through mNGS. Moreover, tNGS outperformed other methods for diagnosing spinal TB and identifying antibiotic-resistance genes in drug-resistant TB.

## Introduction

A spinal infection is a severe form of intervertebral disc infection and adjacent vertebral osteomyelitis caused by various microorganisms. The incidence of spinal infection has been estimated to be 2.4/100000 per year and is increasing due to the aging population, advancements in diagnostic technology, and the growing volume of invasive procedures in recent years (Grammatico et al., 2008; Camino-Willhuber et al., 2022).

Spinal infection is primarily diagnosed on the basis of the patient’s symptoms, imaging findings, laboratory examinations, and microbial culture (Iwata et al., 2019). Although spondylodiscitis is effectively diagnosed with the advent of Magnetic Resonance Imaging (MRI) (Naselli et al., 2022), it is widely accepted that the most critical diagnosis of spinal infection to assist clinicians in developing targeted antibiotic regimens is early identification of causative microorganisms (Esposito, 2016). However, the timely and accurate diagnosis of spinal infections remains a significant challenge for many patients with chest and back pain. Traditional bacterial culture technology has several drawbacks, including long culture time and low sensitivity and specificity (Chen et al., 2022). Furthermore, conventional media is unable to identify *Mycobacterium tuberculosis*, which is common in spinal infections (Khanna and Sabharwal, 2019; Jain et al., 2020). Delayed or failed diagnosis of spinal infection often leads to catastrophic outcomes, including aggravation of infection, severe spinal deformity, spinal cord and nerve damage, paralysis, and increased mortality (Pola et al., 2018). Additionally, empiric broad-spectrum therapy for patients with undiagnosed spinal infections increases the risk of adverse reactions and antimicrobial resistance (Lee et al., 2022).

Recent studies have shown that metagenomic next-generation sequencing (mNGS) technology exhibits key characteristics, including wide pathogen coverage, fast detection, high positive rate, unbiased detection, and effective detection of dead pathogens (Gu et al., 2019; Wensel et al., 2022; He et al., 2023; Kullar et al., 2023). It plays a critical role in the diagnosis of infectious diseases. Previously, a rare case of *Mycobacterium avium* intracellular spinal infection was identified using mNGS at our center (Lv et al., 2023). However, to the best of our knowledge, only a few studies have been conducted on the use of mNGS for spinal infections.

In this study, the electronic medical records of 76 patients with suspected spinal infections who were followed up for two years were retrospectively analyzed. We aimed to determine the sensitivity and specificity of mNGS in detecting pathogens in spinal infections and to compare its efficacy with traditional culture techniques. Moreover, the differences in diagnostic performance between mNGS and targeted next-generation sequencing (tNGS) were determined.

## Materials and methods

### Study design and patient selection

This retrospective study was further carried out after obtaining the approval of the Ethics Committee of Jiangbei District of Southwest Hospital of Army Medical University. We retrospectively analyzed all patients diagnosed with spinal infection between January 1, 2019, and December 31, 2021. The inclusion criteria were as follows: 1) Confirmation of a suspected diagnosis of spinal infection in all patients; 2) completion of mNGS, bacterial culture, and histopathology simultaneously; 3) methods and specimen collection processes were standardized across all cases. The exclusion criteria were as follows: 1) Patients with severe immunosuppressive systemic disease; 2) patients with multiple systemic infections and etiological evidence of more than three types; 3) patients who were followed up for less than two years. The final diagnosis of the patient was made by combining the clinical treatment results, pathological examinations, imaging findings, and laboratory indicators, and the sensitivity and specificity were calculated.

### Data extraction

The following data were collected from all patients: demographic information, clinical symptoms at admission, site of infection, history of tuberculosis (TB), antibiotic interventions, and the presence or absence of pre-infection factors, including diabetes mellitus, glucocorticoid administration, and immunosuppressive drug administration. Additionally, biopsy procedures, time-consuming sample testing, antibiotic administration protocols, and laboratory test data, including routine blood tests, erythrocyte sedimentation rate (ESR), C-reactive protein (CRP), and procalcitonin (PCT) levels, were documented. Finally, the clinical cure rate was recorded.

The standard of cure for spinal infection is as follows: Clinical manifestations: 1) Complete disappearance of spinal pain and normalization of general condition. Signs: Disappearance of back stiffness and no further aggravation of kyphosis. 2) Laboratory examination: Normal ESR and CRP levels. 3) Imaging examination: Bone bridge formation on X-ray film; CT showing the increased density of the adjacent vertebrae around the lesion. 4) MRI showing a non-significant difference in signals between the diseased vertebra and the surrounding normal vertebra.

### Biopsy procedure

Specific biopsy methods were selected based on the preoperative MRI results. For abscesses present behind the laminae, a color ultrasonic-guided puncture was used to extract the abscess or lavage fluid. An endoscopic or C-arm guided percutaneous biopsy was performed for intervertebral, anterior vertebral, and deep psoas muscles. The detailed endoscopic biopsy procedure was as follows. The patient was placed in the prone position on a radiolucent table, and the target vertebral interval was identified using C-arm X-ray fluoroscopy. After local anesthesia, an endoscopic portal of approximately 8 mm was created using a spinal needle targeting the center of the interlaminar space. The endoscope (SPINENDOS GmbH, Munich, Germany) was inserted directly into the infected intervertebral disc space using a cannula. If necessary, some of the upper and lower facet joints can be removed, or laminectomy can be performed using high-speed drilling to perform numerous surgeries. Upon entry into the disc space, all inflammatory tissues suspected of infection, including the paravertebral muscles, necrotic tissues, intervertebral discs, granulation tissue, and disrupted vertebral bodies, were collected under a microscope. Each sample was divided into three parts, and pathogen culture and pathology analyses were performed separately at our hospital.

### Sample DNA extraction

Tissue samples were placed in homogenizing tubes, and 1.2 mL of sterile water was added. The pus samples were inverted and mixed five times, then transferred to 2-mL centrifuge tubes, centrifuged at 10,000 g for 5 min, and the supernatant was discarded. DNA was extracted using the TIANamp Magnetic DNA Kit (DP710-t2, Tiangen, China) according to the manufacturer’s protocol.

### Multiplex polymerase chain reaction

Unlike mNGS, the tNGS assay requires targeted amplification before library construction. The DNA sample, primer working solution, and multiplex PCR reaction mix were mixed, and negative and positive controls were added, followed by amplification. Finally, magnetic beads were purified, and the concentration of amplified nucleic acid was determined using the Qubit quantitative assay.

### Library construction and sequencing

Following Qubit quantification, DNA libraries were prepared using the KAPA Hyper Prep Kit (KAPA Biosystems), according to the manufacturer’s protocol. Quality control was performed using an Agilent 2100, and the DNA libraries were then subjected to single-end 50 bp sequencing using the Dif seq platform (Dinfectome Medical Technology Inc, Nanjing, China), with a target depth of 3 million reads for the targeted workflow and 20 million reads for the metagenomic workflow.

### Bioinformatics analysis

An in-house developed bioinformatics pipeline was used to identify pathogens (Zeng et al., 2022). First, low-quality reads, adapter contamination, repeats, and shorter sequences (< 36bp) were removed to obtain high-quality sequencing data. The sequences of the human hosts were determined by mapping them to the human reference genome (hs37d5) using bowtie2 software (Langmead and Salzberg, 2012). The reads that could not be mapped to the human genome were retained. These were aligned with the microbial genome database for pathogen identification. Our microbial genome database contains the genome sequences of bacteria, fungi, viruses, and parasites (data source: https://www.ncbi.nlm.nih.gov/).

### Interpretation and reporting

**Figure 1:**
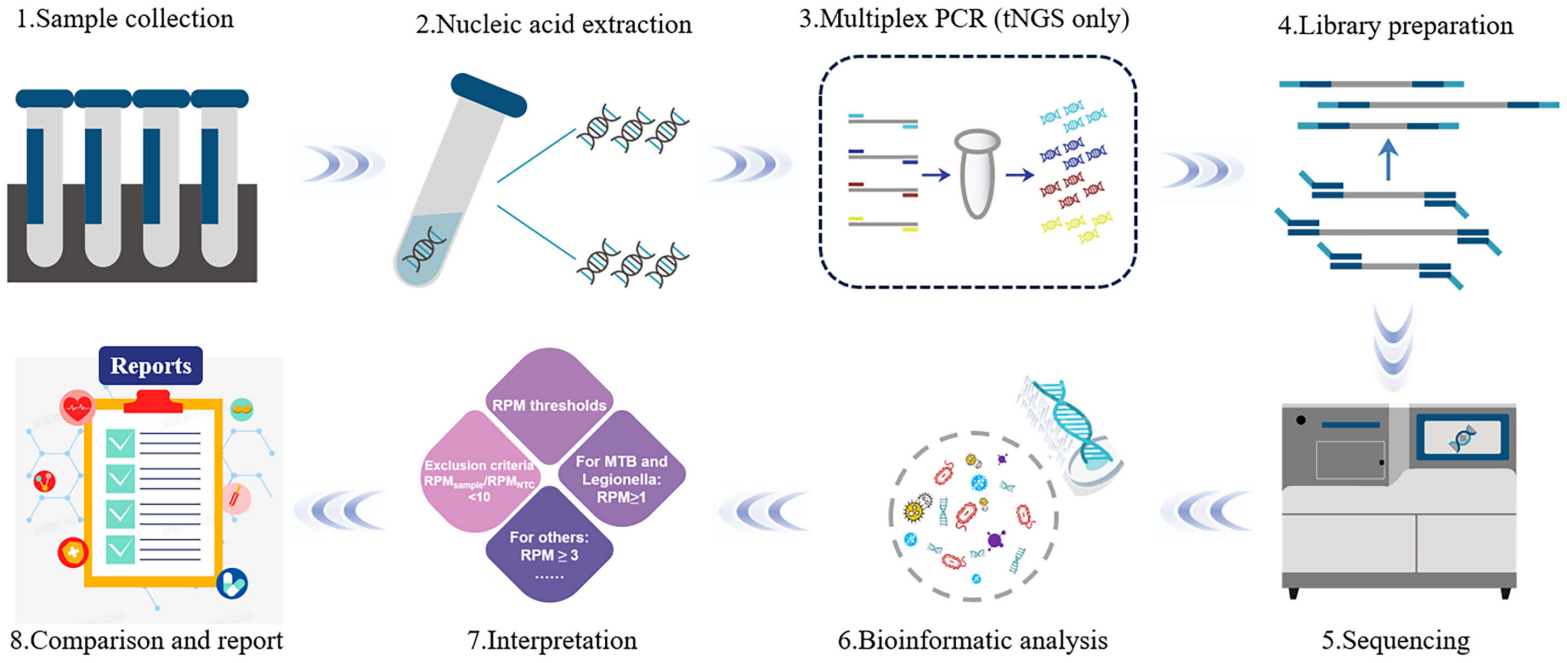
The flow diagram of metagenomic next−generation sequencing technology. The mNGS is mainly divided into two parts: wet lab extraction (1–5) and dry lab pipeline (6–8). Sample collection: Sample collection from the primary site of infection is preferred; 2) Nucleic Acid Extraction: DNA and RNA nucleic acids are extracted using different extraction methods; 3) Multiplex PCR: Unlike mNGS, tNGS requires target amplification before library construction; 4) Library Preparation: Selection of library construction method is based on the sequencing platform and the purpose of the sequencing; 5) Sequencing: The prepared library samples are subjected to single-end 50 bp sequencing on the Dif seq platform; 6) Bioinformatic analysis: based on the analysis of the raw data, the information of species and antibiotic resistance genes in the samples were obtained; 7) Interpretation: Filtering and screening of the detected information according to the series reporting principle; 8) Report: the possible pathogens were screened out according to the analysis results.

The mNGS pathogen detection pipeline was described in previous studies (Miller et al., 2019; Chen et al., 2023; Xu et al., 2023), and the test-positive criteria were as follows: 1) for detection of *Mycobacterium tuberculosis*, *Nocardia*, and *Legionella pneumophila*, at least one species-specific read was required; 2) for other bacteria, fungi, viruses, and parasites, at least three specific reads were required; 3) pathogens were excluded if the ratio of the number of microbial reads per million of a given sample to the NTC was < 10. The sequencing process of mNGS and tNGS is shown in detail in **Figure 1**.

### Statistical analysis

Data are presented as mean ± standard deviation. Continuous variables were compared by an independent sample t-test. The statistical data were described in terms of case numbers and percentages (%) using the χ2 test or Fisher’s exact test. The specificity, sensitivity, positive and negative predictive values (PPV/NPV) of mNGS and culture, and corresponding 95% confidence intervals (CI) were calculated based on histopathological and clinical cure outcomes. SPSS 21.0 software was utilized for conducting statistical analysis (SPSS, Chicago, IL, USA) and statistical significance was determined using bilateral testing with P < 0.05.

## Results

### Demographic characteristics

A total of 76 patients (mean age, 58.70 ± 13.97 years) were enrolled in the study based on clinical symptoms, laboratory tests, and radiological examination. Among them, 11 (14.47%) had diabetes, 11 (14.47%) had hypertension, 11 (14.47%) had a history of TB, and 47 (61.8%) had been exposed to antibiotics before biopsy. The most commonly reported symptoms were back pain (71 cases), fever (25 cases), radiative pain in the lower limbs combined with paralysis (31 cases), nerve dysfunction (10 cases), and American Spinal Injury Association (ASIA) grade D (10 cases). The pathological features included osteonecrosis, neutrophil infiltration, lymphocyte infiltration, chronic granulomatous inflammation, and caseous necrosis. The levels of CRP and ESR before surgery were 39.72 ± 32.68 mg/L and 51.99 ± 29.23 mm/h, respectively. Biopsy was most commonly performed at the lumbar spine level and intervertebral disc site, accounting for 78.95% of total cases (**Table 1**).

**Table 1:** Baseline characteristics and test results of enrolled patients.

Total	Cases	76
Age	Mean ± SD (range)	58.70 ± 13.97(13–88)
Sex	Male (%)	47(61.84%)
	Female (%)	29(38.16%)
Underlying disease	Hypertension (n, %)	11(14.47%)
	Diabetes (n, %)	11(14.47%)
	Hepatitis B (n, %)	4(5.26%)
	Hypothyroidism (n, %)	1(1.32%)
	Cerebral Infarction (n, %)	1(1.32%)
Tuberculosis-related	History of tuberculosis (n, %)	11(14.47%)
Drug intervention before biopsy	Antibiotic n (%)	47(61.8%)
	Nonsteroidal Anti-inflammatory Drugs, n(%)	70(92.1)
Laboratory tests	WBC (×10^9^/L)	6.95 ± 2.45
	HGB(g/L)	114.67 ± 17.24
	PLT (×10^9^/L)	266.74 ± 85.47
	LYMPH (×10^9^/L)	1.30 ± 0.57
	NEUT (×10^9^/L)	4.97 ± 2.38
	LYMPH% (100%)	19.98 ± 9.52
	NEUT% (100%)	67.95 ± 14.68
	CRP (mg/L)	39.72 ± 32.68
	ESR (mm/h)	51.99 ± 29.23
Types of procedures	Endoscopic biopsy (n, %)	11(14.47%)
	Aspiration biopsy (n, %)	48(63.16%)
	surgical biopsy (n, %)	17(22.37%)
Placements of spinal infection	Lumbar vertebra (n, %)	60(78.95%)
	Thoracic vertebra (n, %)	14(18.42%)
	Cervical vertebra (n, %)	2(2.63%)

SD, standard deviation; WBC, white blood cell; HGB, Hemoglobin; PLT, Platelet count; NEUT, Neutrophil; CRP, C-reactive protein; ESR, erythrocyte sedimentation.

### Comparison of mNGS and culture methodology (positive rate and timeliness)

**Figure 2:**
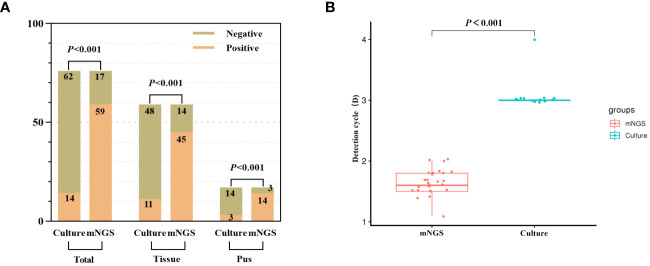
The detection rate of pathogenic microorganism was compared between mNGS and culture. **(A)** The positive rate of mNGS was significantly better than that of culture (total samples, tissue samples and pus samples). **(B)** The detection time of mNGS was significantly less than that of culture.

The pathogen detection rate of mNGS was 77.6% (59/76), whereas that of the bacterial culture was 18.4% (14/76). The difference between the two methods was statistically significant (p < 0.001), with mNGS exhibiting a significantly higher detection rate. To further compare pathogen detection rates, the samples were segregated into pus and tissue samples. The pathogen detection rate of mNGS in infected tissue and pus was 76.3% and 82.3%, respectively, while those for bacterial culture were 18.6% and 17.6%, respectively. Additionally, mNGS exhibited an average pathogen detection time of 1.65 days, whereas bacterial culture had an average time of 3.07 days. The significantly lower average time of mNGS than that of bacterial culture (*p* < 0.001) underscores its timeliness in pathogen detection (**Figure 2**).

### mNGS and culture diagnostic performance in diagnosing spinal infection

**Figure 3:**
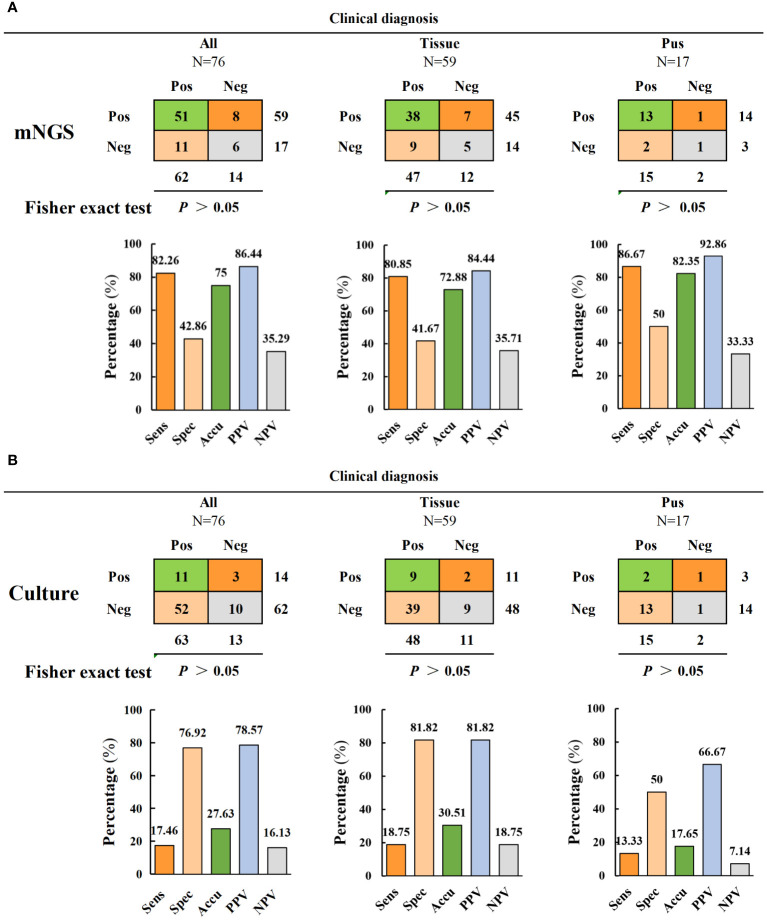
Comparison of diagnostic performance of mNGS and culture. **(A, B)** The diagnostic performance of mNGS and culture includes sensitivity, specificity, accuracy, positive predictive value and negative predictive value (and is divided into two subgroups of tissue samples and pus samples for comparison).

The diagnostic efficacy indices of mNGS and culture are shown in **Figure 3** and **Table 2**. The results revealed significant differences in the sensitivity and accuracy of mNGS compared to conventional bacterial culture. The sensitivity of mNGS (82.3%) was significantly higher than that of culture (17.5%) (*p* < 0.001). Moreover, the accuracy of mNGS (75%) was significantly higher than that of culture (27.6%) (*p* < 0.001). However, there were non-significant differences in specificity, positive predictive value (PPV), and negative predictive value (NPV) between culture and mNGS. The specificity, PPV, and NPV of mNGS were 42.9%, 86.4%, and 35.3%, respectively, while those of culture were 76.9%, 78.6%, and 16.1% (**Figure 3** and **Table 2**). The samples were divided into two subgroups of infected tissues and pus to further compare the diagnostic efficacy. The mNGS results revealed that the sensitivity, specificity, accuracy, and PPV of pus were higher than those of the tissue samples (86. 7% versus 80.9%, 50% versus 41. 7%, 82.4% versus 72.9%, and 92.9% versus 84.4%) (**Figure 3A**). Conversely, the sensitivity, specificity, accuracy, PPV, and NPV for tissue samples were higher than that for pus in the culture group (18.8% versus 13.3%, 81.8% versus 50%, 30.5% versus 17.7%, 81.8% versus 66. 7%, and 18.8% versus 7.1%) (**Figure 3B**). Further analysis revealed that performance comparisons between different sample types were non-significant; however, corresponding trends were observed based on the specific performance data. The diagnostic performance of mNGS was superior in pus samples, whereas bacterial culture exhibited a higher diagnostic value in tissue samples than in pus, suggesting that collecting different sample types may be recommended for different testing modalities in spinal infections.

**Table 2:** The pathogen diagnostic performance of mNGS and culture.

Performance indicators	mNGS(n/%)	Culture(n/%)	χ2	*P* value
Sensitivity	51/82.26	11/17.46	52.4844	<0.001
Specficity	6/42.86	10/76.92	1.9827	0.1591
Accuracy	57/75	21/27.63	34.1289	<0.001
PPV	51/86.44	11/78.57	0.1053	0.7456
NPV	6/35.29	10/16.13	3.0341	0.0815

Clinical diagnosis was used as the gold standard.

### Pathogen spectrum and consistency analysis of mNGS and culture

**Figure 4:**
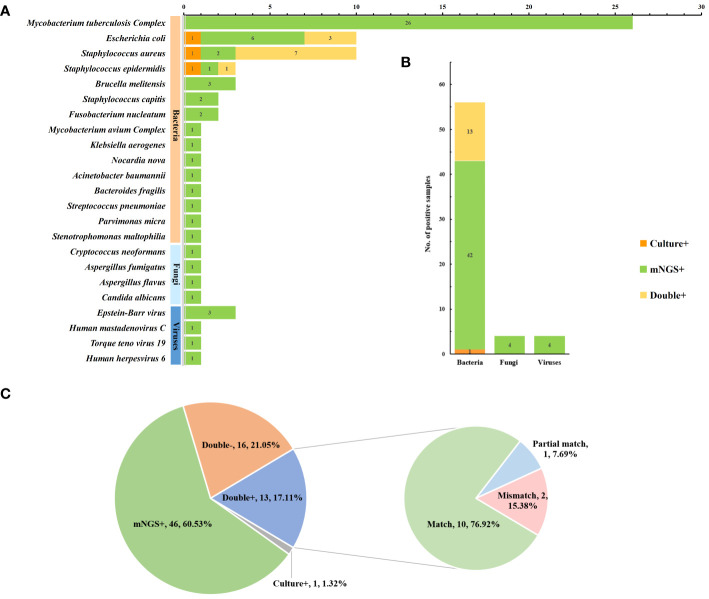
Pathogen spectrum and consistency analysis of mNGS and culture. **(A)** Comparison of pathogen spectra detected by mNGS and culture **(B)** The number of cases of mNGS and cultures detected in different types of microorganisms **(C)** Detect consistency analysis.

The microbial spectra detected using mNGS and culture are displayed in **Figure 4**. *Mycobacterium tuberculosis complex* was the most commonly detected microorganism by mNGS (26 cases), followed by *E*scherichia*. coli* (9 cases), *Staphylococcus aureus* (9 cases), *Staphylococcus epidermidis* (2 cases), and *Brucella melitensis* (3 cases). Conversely, the most commonly isolated microorganisms using culture-based methods were *Staphylococcus aureus* (8 cases), *Escherichia coli* (4 cases), and *Staphylococcus epidermidis* (2 cases). The number of pathogens detected by mNGS and culture at the species level revealed that bacteria were the most common pathogens in spinal infections (56 cases, 73.7%), followed by fungi (4 cases, 5%) and viruses (4 cases, 5%). In the consistency analysis, all 13 cases (17.1%) were positive, and all 16 cases (21.1%) were negative. Among the patients with double-positive results, 10 patients (76.9%) were matched, 2 (15.4%) were partially matched, and 1 (7.7%) was not matched (**Figure 4C**). It is noteworthy that obtaining culture results for TB samples in our hospital was challenging due to the requirement of a P3 laboratory for M*ycobacterium tuberculosis* culture. In this study, 26 samples were further cultured with Roche medium according to mNGS results, indicating a complex infection of *Mycobacterium tuberculosis*; 14 samples were successfully identified as *Mycobacterium tuberculosis*, and 3 were identified as multidrug-resistant TB.

### Comparison of diagnostic performance between mNGS and tNGS in tuberculosis patients

**Figure 5:**
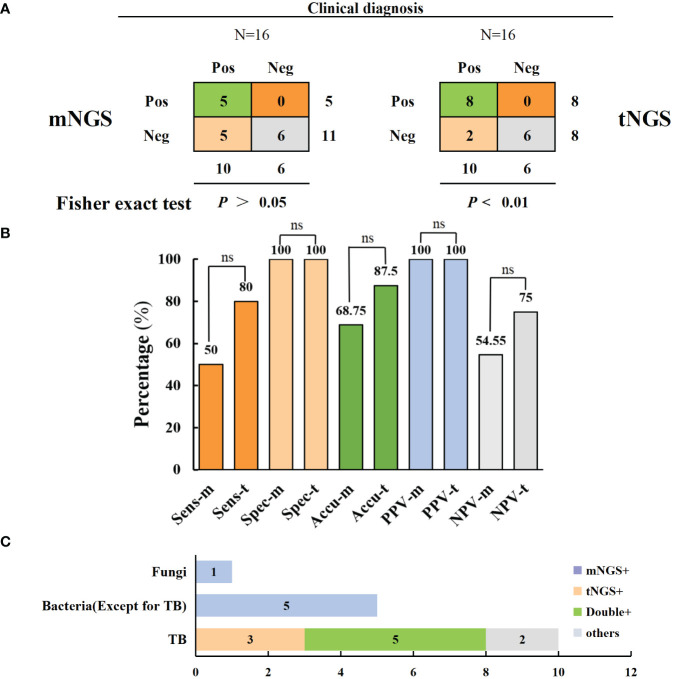
Comparison of mNGS and tNGS in the diagnosis of spinal tuberculosis. **(A, B)** In comparison of the diagnostic performance of mNGS and tNGS, tNGS has a higher diagnostic sensitivity for tuberculosis (no statistically significant difference) **(C)** mNGS has the advantage of additional diagnosis of bacterial and fungal infections. ns: no significance.

A total of 16 samples underwent both mNGS and tNGS simultaneously; 10 of them underwent culturing, and 4 were positive. Based on pathological examination and clinical outcomes, 10 patients were clinically diagnosed with TB. Consequently, tNGS can be compared with mNGS to evaluate the sensitivity of targeted sequencing technology for TB diagnosis. The results indicated that tNGS demonstrated higher sensitivity and accuracy in diagnosing TB than mNGS (80% versus 50% and 87.5% versus 68.8%) but without significant difference. There was a significant correlation between the tNGS detection results and clinical diagnosis (*p* < 0.01), whereas a non-significant association was observed between the mNGS detection results and clinical diagnosis (*p* > 0.05). However, it is noteworthy that mNGS has a broader coverage and can be used for diagnosing bacterial and fungal infections (**Figure 5** and **Table 3**). Moreover, three cases of resistant TB were identified in 16 patients using tNGS, with the resistance genes rpoB, pncA, and rpsL, whereas no resistance genes were detected using mNGS. tNGS outperformed mNGS in detecting resistant TB.

**Table 3:** Detection of mNGS other than Mycobacterium tuberculosis in six samples.

Case	mNGS	tNGS(TB/NTM)
Case 1	*Escherichia coli*	/
Case 2	*Staphylococcus aureus*	/
Case 3	*Aspergillus fumigatus*	/
Case 4	*Mycobacterium tuberculosis Complex; Streptococcus pneumoniae; Parvimonas micra*	*Mycobacterium tuberculosis Complex*
Case 5	*Staphylococcus epidermidis*	/
Case 6	*Brucella melitensis*	/

## Discussion

Spinal infections pose a complex challenge for spinal surgeons regarding diagnosis and treatment. These infections are caused by the transmission of microorganisms through the bloodstream from distant infected sites, commonly by bacteria, including *Mycobacterium tuberculosis*, S*taphylococcus*, and *Escherichia coli* (Huang et al., 2023). Effectively treating spinal infections involves identifying the pathogenic microorganism and selecting the appropriate antibiotic based on the microorganism’s drug sensitivity, while carefully considering antimicrobial sensitivity and bone tissue permeability. Conservative treatment with antibiotics, bed rest, and spinal bracing is successful for most patients with spinal infections (Lener et al., 2018). However, diagnosing spinal infections can be difficult due to the small quantity of samples obtained, resulting in a low positive rate of puncture bacterial culture (Wang et al., 2023; Zhang et al., 2023a). Additionally, the proximity of the infection to the spinal cord poses a challenge. Furthermore, the limited availability of facilities to conduct drug susceptibility tests for M*ycobacterium tuberculosis* leads to empirical treatment, potentially resulting in treatment failure and an increase in acquired drug resistance. Therefore, improving the routine etiological diagnosis of spinal infections is imperative. Recent studies have demonstrated the potential of mNGS to accurately diagnose various infections, including spinal infections. In 2014, the New England Journal of Medicine reported, for the first time, the use of mNGS to accurately diagnose intracranial leptospirosis infection, while 38 conventional tests were negative (Wilson et al., 2014). In 2021, Fang et al (Fang et al., 2021). obtained more microbial information through mNGS detection in patients with periprosthetic infection after total joint replacement, and optimized culture methods to improve diagnostic efficiency and the positive rate of drug susceptibility tests. In 2023, Wang et al. used mNGS in 25 patients with spinal infections and concluded that the sensitivity of mNGS was higher than that of bacterial culture (Wang et al., 2023). In this study, 76 patients with suspected spinal infections were included, and a two-year follow-up was conducted to assess the effectiveness of mNGS. The study combined clinical treatment outcomes, pathological examination, imaging changes, and laboratory indicators to minimize biases like infection recurrence and missed diagnoses. The results revealed that mNGS exhibited significantly higher sensitivity than bacterial culture, albeit with lower specificity, and highlight the potential of mNGS in detecting pathogens in spinal infections. Moreover, mNGS exhibited a higher diagnostic value in pus than in tissue, with higher sensitivity and specificity; conversely, the sensitivity and specificity of bacterial culture were higher for tissue than for pus. Although there were non-significant differences, the specific performance data revealed these trends. These results indicate that the bacteria in pus are more likely to be dead, and the nucleic acid sequences of dead bacteria still play an irreplaceable role in mNGS. Contrarily, the relatively rich blood supply in infected lesion tissues facilitates bacterial growth, leading to a higher proportion of viable bacteria and a higher diagnostic value in bacterial culture. Therefore, the site selection for spinal biopsy samples should be further standardized. It is recommended to prioritize pus samples for mNGS testing, while for culture testing, it is recommended to select samples of infected lesions and borderline areas of normal tissue.

Furthermore, the detection duration of mNGS was significantly shorter than that of conventional culture, indicating that chemotherapy regimens were available earlier to control infections that could not be managed surgically. Additionally, the culture methods could be adjusted according to the results of mNGS to improve the positive rate of culture and clear antibiotic sensitivity and reduce the misdiagnosis rate of single mNGS detection (Fang et al., 2021). Several studies have demonstrated that mNGS is unable to differentiate among strains with high homology, which may lead to errors in strain identification (Simner et al., 2018; Li et al., 2021). Similar to previous studies, *Mycobacterium tuberculosis*, *Staphylococcus aureus*, and *Escherichia coli* were the most common pathogenic microorganisms in spinal infections. However, it is noteworthy that these data are not indicative of the common bacterial profile of spinal infections because this study was conducted at a single center. Moreover, mNGS exhibits high sensitivity and a wider detection range, which can lead to a higher false-positive rate than traditional detection methods. Viral infection was detected in four patients in the pathogen spectrum; however, a comprehensive analysis of pathological examinations and laboratory detection resulted in false positives. In contrast to previous studies reporting the poor performance of mNGS in diagnosing fungal diseases, this series successfully confirmed four cases of fungal infection using mNGS, even when culture results were negative. However, the notion that mNGS is more sensitive to fungal infections than traditional detection methods should be interpreted with caution. Theoretical analysis and literature review indicate that false-negative results of mNGS in fungus detection remain difficult to overcome. This is primarily due to the thick fungal cell wall, which makes DNA extraction difficult, resulting in low nucleic acid concentrations that could be easily missed, and the high host DNA background, which reduces the sensitivity of mNGS to detect fungal infections.

mNGS application for pathogen detection in clinical samples is limited by the high proportion of human nucleic acids, which reduces the sensitivity and increases the risk of missing drug resistance and virulence genes. To address this limitation, tNGS combines ultra-multiplex PCR and high-throughput sequencing to target specific pathogen sequences, leading to improved detection of microbial sequences, coverage, and the ability to identify virulence and drug resistance genes (Ballester et al., 2016; Wu et al., 2023). This approach has proven to be particularly advantageous for the diagnosis of TB. Murphy et al. (Murphy et al., 2023) demonstrated that tNGS can accurately predict drug resistance in *Mycobacterium tuberculosis* obtained from clinical specimens or cultures. Sibandze et al. (Sibandze et al., 2022) reported that tNGS detected *Mycobacterium tuberculosis complex* DNA in 68% of stool samples from TB patients, with a complete drug resistance prediction report obtained for 74% of the detected MTB complexes. They emphasized the importance of widespread tNGS testing to combat drug-resistant TB. Moreover, Zhang et al. (Zhang et al., 2023b) highlighted the potential of tNGS in identifying spinal TB and predicting drug resistance, achieving a 100% detection rate for *Mycobacterium tuberculosis*, and identifying antibiotic resistance genes and drug-resistant mutations in 18 patients. A comparison of mNGS and tNGS in 16 patients with suspected spinal TB confirmed that tNGS exhibited higher sensitivity, accuracy, and NPV with non-significant differences. This might be attributed to the small sample size, with only 16 patients undergoing mNGS and tNGS simultaneously. The sample size may be increased to validate these results further. Furthermore, tNGS identified three cases of spinal drug-resistant TB, one of which exhibited rifampicin resistance due to the rpoB mutation, consistent with previous studies demonstrating that rifampicin resistance is prevalent and associated with the rpoB gene (Zhang et al., 2023b). This implies that tNGS can be used to diagnose spinal TB and predict drug resistance. However, tNGS has limitations in detecting unknown pathogens, and the number of detected pathogens remains lower than that of mNGS.

Our study has several limitations. First, this retrospective study with an inherently low level of evidence did not include a matched control group; compared to traditional open debridement, the advantages of mNGS in the diagnosis and treatment of spinal infection patients have not been confirmed. Second, the relatively small sample size associated with extremely low drug-resistant microorganisms makes further comprehensive analysis difficult to determine the advantages of tNGS in detecting antibiotic-resistance genes in drug-resistant bacteria. Moreover, the relatively short follow-up time may have confounded the clinical results, indicating that the infection may recur after antibiotic discontinuation as an intracellular infection in TB patients. Additionally, *Mycobacterium tuberculosis* cultures were performed for all patients in specialized TB hospitals after the mNGS results indicated possible TB infection. Notably, the sensitivity of cultures may have been reduced to some extent due to this process, potentially impacting the accuracy of the results. The culture time for TB bacteria is typically around 30 days. Therefore, it is possible that the culture time in this series may not reflect the actual data, potentially leading to discrepancies in the results.

## Conclusion

Compared to culture, mNGS exhibited significantly higher sensitivity, accuracy, PPV, and NPV for spinal infection and demonstrated better diagnostic value in developing an antibiotic regimen earlier. Furthermore, it is recommended to prioritize pus samples for mNGS testing procedures while samples of infected lesions and borderline areas of normal tissue for culture. Moreover, tNGS demonstrated overwhelming superiority in diagnosing spinal TB and identifying antibiotic-resistance genes in drug-resistant TB. However, both mNGS and tNGS have limitations, including lower specificity compared to culture, higher false-positive rates for mNGS, and difficulties in detecting fungi and intracellular bacteria for tNGS. Therefore, it is important to use a combination of multi-detection methods to improve the accuracy of diagnosis and clinical outcomes.

## Data availability statement

The original contributions presented in the study are included in the article/supplementary material. Further inquiries can be directed to the corresponding authors. The data presented in the study are deposited in the SRA repository, accession number PRJNA1087060.

## Ethics statement

The studies involving humans were approved by 958th Hospital of the PLA Army. The studies were conducted in accordance with the local legislation and institutional requirements. The participants provided their written informed consent to participate in this study.

## Author contributions

HL: Data curation, Methodology, Software, Writing – original draft, Writing – review & editing. SL: Data curation, Software, Writing – original draft. ZS: Data curation, Formal analysis, Software, Writing – review & editing. YG: Data curation, Formal analysis, Writing – review & editing. JZ: Data curation, Formal analysis, Writing – review & editing. HC: Data curation, Writing – review & editing. FL: Conceptualization, Methodology, Visualization, Writing – review & editing. JX: Conceptualization, Investigation, Methodology, Project administration, Supervision, Visualization, Writing – review & editing. ZrZ: Conceptualization, Formal analysis, Funding acquisition, Investigation, Methodology, Project administration, Software, Supervision, Validation, Writing – review & editing. ZhZ: Conceptualization, Data curation, Formal analysis, Funding acquisition, Investigation, Methodology, Project administration, Supervision, Visualization, Writing – review & editing.
